# The ‘Molecule of the Month’ Website—An Extraordinary Chemistry Educational Resource Online for over 20 Years

**DOI:** 10.3390/molecules22040549

**Published:** 2017-03-29

**Authors:** Paul W. May, Simon A. Cotton, Karl Harrison, Henry S. Rzepa

**Affiliations:** 1 School of Chemistry, University of Bristol, Bristol BS8 1TS, UK; 2 School of Chemistry, University of Birmingham, Edgbaston, Birmingham B15 2TT, UK; s.cotton@bham.ac.uk; 3 Department of Chemistry, University of Oxford, South Parks Road, Oxford OX1 3QZ, UK; karl.harrison@chem.ox.ac.uk; 4 Department of Chemistry, Imperial College, South Kensington Campus, London SW7 2AZ, UK; rzepa@imperial.ac.uk

**Keywords:** online chemistry resources, chemistry education, chemical databases

## Abstract

The Molecule of the Month website (http://www.chm.bris.ac.uk/motm/motm.htm) is an educational resource that is celebrating its 20th anniversary. Here we reflect on its pioneering role in promoting new technology for visualizing and presenting chemical information on the web, as well as its achievements, as a free educational resource, both as a teaching aid and as a multi-user, multi-author learning platform. We discuss the legal aspects of such sites, as well as issues around how to make the content permanent. Finally, we look forward to how such sites may evolve in the future.

## 1. Introduction

**Figure 1:**
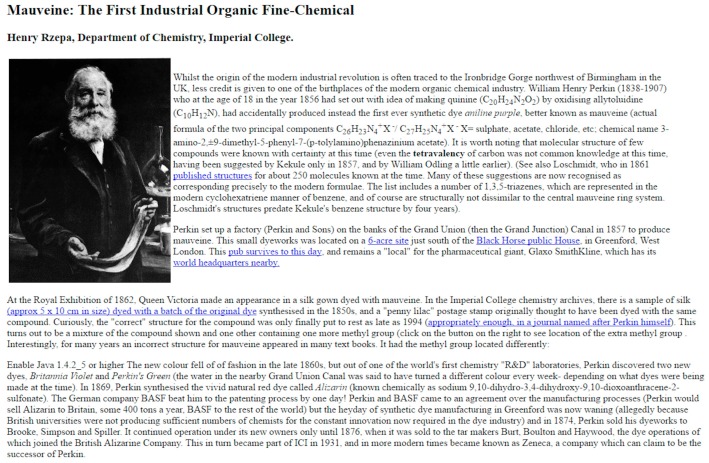
Screenshot of the first MOTM page for January 1996 about mauvine dye [4].

The Internet/World Wide Web has been with us for just over two decades, and it is commonplace to say that it has revolutionized the ways in which we communicate with each other. The way chemical information is created, shared, disseminated, and managed has also changed significantly over that time [1]. Just over 20 years ago, a group of chemistry academics in the UK were thinking about ways to use these new media to communicate the wonder of chemistry to a wider audience. The original idea came from Henry Rzepa of Imperial College London, who brought together Karl Harrison of the University of Oxford and Paul May of the University of Bristol to try to produce regular monthly articles showcasing important chemical substances and to host them on their department’s website. This coincided with, and was in part driven by, the arrival of new browser software that allowed 3D molecular structures (then called ‘hyperactive molecules’) [2] to be displayed interactively on a webpage. The central website linking these articles together became known as the Molecule of the Month (MOTM) website [3], with the first article about the molecule mauveine (see Figure 1) appearing in January 1996—which predates many university Chemistry department websites!

The original idea was that the three founders would each write only the occasional MOTM article; most of the content (it was hoped) would be written by willing volunteers from around the world. This was broadly true for the first 10 years or so, with contributions coming from a whole range of authors drawn from across the globe. Most came from the university sector, but some were written by chemists in industry and some by schoolteachers and students, the youngest being 15-year-old Layth Hendow, of Hull Collegiate School, Yorkshire, who wrote about Teflon in June 2009 [5]. Some of the earliest MOTM articles were short and rather basic [6]. However, the more recent ones have been quite detailed essays, with scientific diagrams, videos, and synthetic reaction schemes—more reminiscent of mini-review papers than web pages.

Other ‘molecule’ sites followed in the wake of MOTM, such as one based at Virginia Commonwealth University, and another at Prous in Spain, but gradually many of these have fallen by the wayside. Others have grown up in or morphed into the blogging sector [7,8,9,10], while the ACS has a Molecule of the Week site [11].

## 2. Legal Worries

One of the issues that began to emerge around the year 2000 was that of legal liability for the content of the sites. At the time, the three founders were eagerly persuading undergraduate chemistry students at their universities to write small MOTM-style pages as web projects (originally sponsored with prizes by the Royal Society of Chemistry (RSC) as part of their Exemplarchem project), the best of which were then chosen to become an official MOTM article soon after. Cajoling students to do this was surprisingly easy, as that time it was the height of the ‘dotcom boom’ and so many undergraduates were keen on learning about the web and creating content for it. An example is the MOTM page in May 2001 about capsaicin, written by the then Bristol undergraduate Matt Bellringer [12]. The students who created these webprojects said they found the experience very fulfilling, in that it was unusual within a science degree to have an opportunity to do something creative. Researching, writing, and then designing a colourful MOTM-style webpage that encompassed scientific ideas in a visually attractive medium was a novel experience to many students—and for some it even inspired them to alter their career path in favour of scientific writing, publishing, or even outreach.

However, the issue that began to worry us was that, because the three founder universities were hosting the MOTM webpages on their webservers, they were effectively publishing scientific documents that had been written by students—and because these had not been peer-reviewed or sometimes not even thoroughly fact-checked by an academic, the articles were potentially full of errors. Criticism came from a few of the more old-fashioned chemistry academics who did not think it was appropriate for student-generated content, especially about such indiscreet subjects as Viagra and absinthe, to be publicly available on an official university site. More importantly, the university lawyers began to worry whether the universities hosting the MOTM pages might be laying themselves open to litigation. An example might be if someone read a factually incorrect MOTM article written by a student, and then acted on this false information causing harm to themselves or others. For instance, if a reader tried to follow an incomplete synthesis recipe that led to an accident, or ingested a poisonous substance that the MOTM page had assured them was non-toxic. In the light of these worries, all the MOTM pages were removed from the Oxford server, and hosted instead on an independent server operated by the company 3dchem.com [13]. The subsequent content was all written and verified by an academic (Karl Harrison), who transferred all the files to a database and then dynamically generated the web content from these files and an HTML template. This gave those web pages a well-designed and consistent style, and this approach is exemplified now by *WordPress* and many other Content Management Systems (CMS) used by web authors today.

The original MOTM site hosted at Bristol University, however, carried on regardless, except that the content became much more carefully vetted. In fact, the only MOTM page that was ever withdrawn was one about sarin nerve gas (March 1999) because it contained rather too much detail about the precursors and the synthesis procedure than was deemed expedient—even though those details were freely available in the scientific literature (and still are available on Wikipedia and the wider internet).

## 3. Pioneering Innovation on the Chemical Web

**Figure 2:**
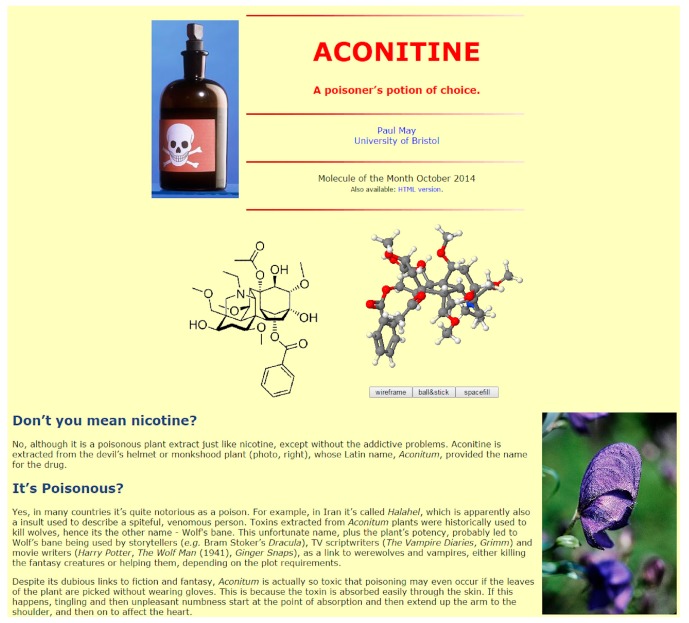
The MOTM page for October 2014 was about the poison aconitine [18]. The JSMol version includes both the 2D structure (on the left) and the interactive 3D structure (on the right).

One could argue that the ground-breaking combination of accessible scientific content and interactive visualization software found on the MOTM website (and others like it) helped to drive innovation in the chemical web. In the mid-1990s, the potential to make a chemistry web page ‘come alive’ with rotatable interactive 3D molecular structures inspired developers at MDL to convert the existing molecular visualization program Rasmol into the web-browser plug-in Chemscape Chime [14]. Chime quickly became the mainstay of the early MOTM pages, and helped to promote the web as a new and fun educational resource for schoolchildren and university students alike. Other visualization plug-ins quickly followed, such as Cortona3D [15] (which used 3D VRML structure files), and the JMol viewer [16], which arrived in 2003 and did a similar job but via Java applets running on the browser. As time passed, many of these innovations that accompanied the early web, including Chime and Cortona3D, faded into history. JMol remained popular until about 2013; however, because it relied on the browsers having Java installed, and on the users allowing the applet to run (thereby ignoring all warnings from the browser that such external applets might be dangerous), meant that this, too, became difficult to implement. JMol has recently been superseded by JSMol [17], which runs a much safer version of Javascript in the browser (without scary pop-up warnings), but with all the same functionality and 3D interactivity as before. All new MOTM pages now have an optional JSMol version (see, for example, Figure 2) and many of the older articles are gradually having a JSMol version ‘retrofitted’.

Despite the MOTM site being one of the pioneers of new visualisation technology for webpages with chemical content, standard HTML versions of articles, containing only text and images, have always been at its core. This meant that, even though some casual readers might not have had Chime or Java installed to help them visualise some of the chemical structures, there has always been an HTML-only version readable by all.

It could be argued that the MOTM site had other, more general, influences on the modern chemical web. Back in 1996, the semantic web had not yet been born, and it would take several more years before it began to be developed for chemistry [19]. Even so, some of the MOTM articles already had semantic information embedded in them. Thus, the HTML in the first article [4] already contained links to semantic expressions of the molecules in the form of XML documents (also called datuments to indicate their data-rich character) [20], with further metadata present in the form of the InChI molecular identifier acting as potential nodes in an RDF semantic graph [21]. When this particular MOTM instance morphed into a Wordpress hosted blog [10], further semantic exposure was added using plugins such as ‘Dublin Core for Wordpress’ or ‘wp-RDFa’ helping to express metadata. It would be fair to say that the MOTM sites provided useful early testbeds for tracking the evolution of the semantic web.

Another example was the evolution of some online chemical databases such as the Cambridge structure database (CSD) [22] into having one ‘landing page’ (as it is now called) per entry identified by the DOI persistent identifier. This formally exposed metadata for the entry, which was aggregated using the DataCite resource [23]. Unfortunately, the original MOTM articles do not yet have allocated DOIs allowing such metadata exposure (ref. [4] is the sole current example). The development of a Wordpress plugin supporting the DOI system is also still in the future as this article is written; both these aspects are discussed in more detail below. The MOTM page also paved the way into the modern area of research data management (RDM) [24] and helped to highlight the need for students and postgraduates to be suitably trained in these areas. The MOTM site has even had an impact upon how some journals are published. Shortly after the first MOTM articles appeared, the American Chemical Society introduced something they call WEO (Web Enhanced Objects) [25], which are in fact mini MOTM-style articles embedded into a journal paper as a figure or a table. These WEOs are now starting to be hosted independently of the journal itself in a digital repository. For example, ref. [26] is a MOTM-style WEO/Table hosted on a data repository and supporting a main article published elsewhere [27].

Another pioneering aspect of the MOTM page was that it was one of the first to espouse open content for molecular information and structures; nowadays, this is called FAIR (findable, accessible, interoperable, and re-usable) data [28]. The MOTM articles have always preserved ‘data integrity’, which is to say that the reader could normally obtain the 3D molecular coordinates from a MOTM article (even the HTML-only versions) by one method or another, usually by a direct hyperlink to the coordinate (mol, pdb, or XML) file. Nowadays, however, some publishers of online journals are using ‘active’ versions of viewers or software such as Acrobat 3D that can include such 3D models, but, importantly, in a way that the original and re-usable data (e.g., the molecular coordinates) are rarely extractable. This, of course, is the direction publishers are taking to protect their content (as part of digital rights management, DRM) and to make the experience proprietary. Although this may present the reader with a visually seamless document, they have veered away from the principles established by MOTM, i.e., these documents do not present FAIR data to the reader.

## 4. The Challenge of Wikipedia

The emergence of Wikipedia [29] in 2001 was the first big challenge to the MOTM site and to others like it. Wikipedia has rapidly developed into a free repository of factual information about everything imaginable, including chemicals and molecules. Around the same time, online databases of spectroscopic and structural data (such CSD [22] and SDBS [30]) and searchable molecular structures [31,32,33] also became readily available. Not long after Wikipedia and these other online chemical resources became established, the MOTM site experienced a large drop in hits, along with a reluctance of authors to write and contribute new MOTM pages. After all, why would anyone bother reading (or writing) a MOTM page when all the facts you could ever possibly want about every molecule are already on Wikipedia?

**Figure 3:**
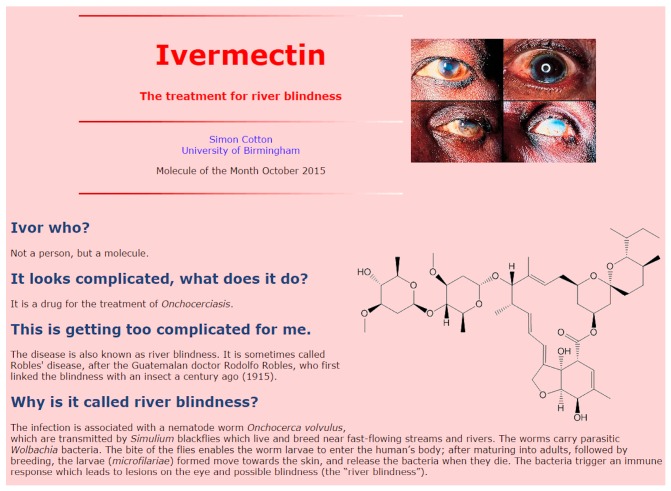
The Q&A style used in most MOTM pages, including this one about ivermectin from October 2015 [35].

Originally, MOTM articles often featured research advances of interest to the authors, and the style could be rather academic. What was needed was a change of style, to differentiate a MOTM article from a Wikipedia entry. This revamp was brought about by Simon Cotton’s first MOTM entry about tetraethyl lead in January 2001 [34]. The innovation was to write the article in an informal question-and-answer style, which grouped the information up into a series of short paragraphs in the form of a curious student asking questions of a knowledgeable, patient, but long-suffering teacher. Simon’s background as a ‘long-suffering’ schoolteacher was ideal for this, and this style introduced a breath of fresh air into the rather ‘textbooky’ format of previous MOTMs. Nearly all the MOTMs nowadays use this Q&A style (see Figure 3, for example), and the MOTM articles have continued to be posted monthly—not a bad achievement after 20 years!

## 5. The MOTM Site as an Educational Resource

The MOTM site compasses a vast range of themes, found everywhere from foodstuffs to medicines and from explosives to toxins. It exists to convey a little of our contributors’ excitement to a wide readership. We do not just concentrate on the facts, but try to weave an interesting story about the history of that month’s molecule, its role in modern society (including references to rock/pop music, Hollywood films/filmstars, TV programmes, historical figures, etc.), and its good and bad points. For controversial molecules (DDT, thiomersal, bisphenol A, etc.), we try to remain neutral, tell both sides of the story, and let the reader make up their own mind. After all, molecules are morally neutral until humans start to interact with them or use them. Communicating chemical ideas in such an informal but engaging manner is entirely consistent with recommendations by chemical educators [36].

Educational buzzwords currently in vogue in UK schools are to ‘stretch and challenge’ students, possibly arising as a reaction to accusations that in recent times schools have been too guilty of spoon-feeding students, i.e., teaching them only to ‘pass the test’. Perhaps as a reaction to that, we have had many MOTM submissions from high-school students (and their teachers), and we have been told by many teachers that they use the MOTM site as a source of inspiration for projects in their chemistry lessons. For students to write a MOTM is a valuable learning experience—readers will know that often the test of one’s understanding is to have to explain that topic to another person. Indeed, one of our regular contributors, Michael Thompson (from Rugby School, UK), who is a very experienced schoolteacher, has written several MOTM articles, both by himself and in collaboration with his sixth-form pre-university students (aged 16–18). Benefitting from the experience of writing a MOTM, one of his student co-authors (Hugh Campbell) went on to set up a student-led Society (‘The Blue Bunsen’), with regular meetings where students presented papers on advanced chemical topics at a post-school level.

We have also had numerous reports from schools that tell us that they regularly use MOTM articles in their schemes of work, and use it as an online resource to complement what is found in textbooks and promote independent learning. With this in mind, we try to aim at a level that is readily understood by a motivated 18-year old. Experience (and feedback) tells us that many adults from a non-scientific, even arts background, find interesting content that they had not expected to encounter. As chemists, we understand that molecules are fascinating in terms of what they do, and we try to share that ‘sense of wonder’, as Deanna Cullen calls it [37], with scientific and non-scientific audiences alike.

## 6. The Issue of Permanency

Even within the first five years of the website, we had started to notice that articles contributed by external authors and hosted on their webservers were not as permanent as we had hoped. Old MOTM articles would disappear, either because in the years since the article had been written the host server had been replaced, or the author had moved jobs, or in two cases because the authors had died and their webpages (and our precious MOTM article) deleted. Luckily, we had made local backups of these pages and thus simply switched the link. However, it made us worry about the permanency of the MOTM project. After 20 years, we have nearly 250 MOTM articles, which include a wealth of data and information that would be a shame to lose, if say, at some future date Bristol University decided it no longer wished to host the site. One solution to this permanency issue, which we are currently investigating, is to retrospectively go through all the MOTM pages and assign each of them a digital object identifier (DOI) [38]. The reason for this is that a DOI is issued in exchange for metadata (which could, for example, include the InChI [39] or SMILES [40] strings/keys for each molecule) that will probably outlast any specific web page. Having a DOI also records ‘impact statistics’ or web-hits independently from the web site.

As another solution to get around this legacy issue, we decided to publish our favourite 60 or so MOTMs in a more long-lasting format—a paper book! Selected articles were rewritten (in the Q&A style) to make them more accessible to a general audience, with less detailed chemistry (synthetic recipes, etc.) and more interesting anecdotes, ‘fun facts’, cartoons, and pictures, and these were collated and published in a book called ‘Molecules that Amaze Us’ [41]. We were originally concerned that people would not buy a book if a large part of its content was already freely available online, but we need not have worried. It seems that the book has actually inspired readers to come back to the website, with hits increasing from 2500 to nearly 10,000 per month in the two years since the book was published. Therefore, it has turned full circle—the website inspired the book, which inspired people to view the web site.

## 7. The MOTM Site as a Means to Communicate Chemistry to a (Much) Wider Audience?

Universities and chemists in general are increasingly concerned about communicating their message to the wider world, through media such as outreach and public engagement [42,43]. However, there has been concern for some years that a high proportion of research papers are rarely read or cited, making negligible contributions to knowledge [44], let alone to educating the public. To quote from Meho’s article in Physics World [45]: ‘It is a sobering fact that some 90% of papers that have been published in academic journals are never cited. Indeed, as many as 50% of papers are never read by *anyone* other than their authors, referees, and journal editors.’ This may simply be because, as more journals have become available online, proportionately fewer have been cited [46] (although this has been challenged) [47]. Nevertheless, this highlights the fact that a journal is not the place to publish articles that deserve a large audience.

If a large readership is wanted, ‘scientists now need to make it their job to disseminate their work on as many platforms and in as many different ways as possible’ [45]. Compare the figures above to the number of hits from the MOTM site. In 20 years, the Bristol MOTM website has been read over 4 million times, which is, on average 200,000 times a year, indicating an average readership for each individual MOTM article of 20,000—far more than any of our journal scientific papers could ever achieve. Furthermore, it should be borne in mind that half of those MOTM articles have only been available for 10 years or less.

Another example of the unexpected ways in which MOTM articles have proved useful in the public domain is the molecule acrylamide, a potential carcinogen, whose MOTM entry [48] was written in August 2016 at the time of concern about high acrylamide levels in certain foods. When the subject surfaced again in the UK popular press in January 2017 following the UK Food Standards Agency’s campaign warning people of the danger of cooking starchy foods at high temperatures for long periods, the existence of the MOTM article for acrylamide [48] proved to be a goldmine for journalists wanting accessible information and contacts. Within hours, the article author (Simon Cotton) had appeared on national television news and had also given two long interviews over the telephone, one with a major national newspaper, the other with a grocery trade magazine. This meant a good deal more news exposure for the university in one day than would arise from most journal papers in many years! Similarly, Karl Harrison’s MOTM-style pages at 3dchem.com [13] led to several international conference invites. He was also asked to contribute to the 2008 exhibition ‘Beyond Measure—Conversations across Art and Science’ [49] in Cambridge UK, which then led to the science-inspired gifts as part of the Molecules range in the London Science Museum Shop.

## 8. Looking Ahead

Where will the MOTM project go in the next 10 years? There are still plenty of interesting molecules to write about, and judging from the number of hits, the readership still seems keen to read about them. New technologies, such as augmented reality and virtual reality, are now being adopted by gamers, so perhaps this will be the next innovation to hit MOTM web pages, although they have a fair way to go yet [50]. Virtual-reality modelling language (VRML) was touted in the mid-1990s as a method of creating virtual navigable 3D molecular worlds [51,52], but the idea never really took off, being superseded first by JMol and now by JSMol as the preferred method for constructing virtual molecular models. Nowadays, however, VRML has found a new lease of life for 3D printing. Thus, tactile and colourful 3D-printed molecules and associated properties such as molecular orbitals are increasingly being used as teaching tools [53]. With the 3D coordinate files being downloadable from the relevant MOTM page and readily converted into VRML format with online applications, it is easy to imagine how future school lessons may involve reading about a molecule—then making a model of it to take home.

**Figure 4:**
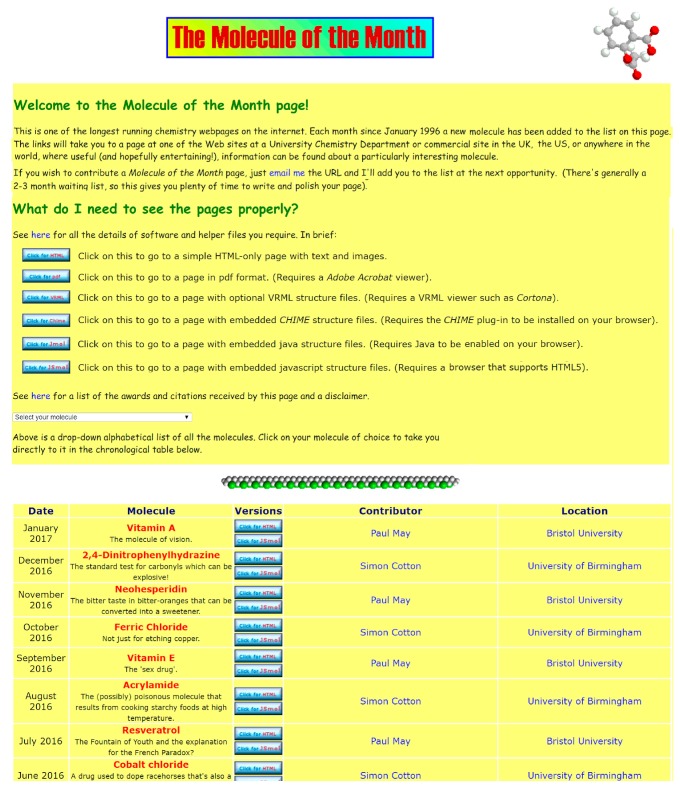
The landing page of the MOTM website [3] as it appeared in January 2017.

The original goal of having the MOTM content written by numerous external contributors did not really pan out. Nowadays, only a couple of MOTM articles per year are written by volunteers, with the remainder being written alternately by two of the authors of this article (see Figure 4). However, perhaps as chemistry education in schools become increasingly more Internet-based, we may yet receive submissions from students and pupils who have been, and continue to be, inspired by the 20 years of chemistry on the web!
